# Supplementary material for: Carotenoid binding in Gloeobacteria rhodopsin provides insights into divergent evolution of xanthorhodopsin types
Source: Commun Biol. 2022 May 30;5:512. doi: 10.1038/s42003-022-03429-2 (PMC9151804; doi:10.1038/s42003-022-03429-2)
Supplement: Supplementary file 2 — Supplementary Information [file 42003_2022_3429_MOESM2_ESM.pdf]

Carotenoid binding in *Gloeobacteria* Rhodopsin provides insights into divergent evolution of xanthorhodopsin types.

Kimleng Chuon[1¶], Jin-gon Shim[1¶], Kang Kun-Wook[1], Shin-Gyu Cho[1,2], Chenda Hour[1], Seanghun Meas[1], Kim Ji-Hyun[1], Ahreum Choi[3], Kwang-Hwan Jung[1, *]

[1] Department of Life Science and Institute of Biological Interfaces, Sogang University, 35 Baekbeom-Ro, Mapo-Gu, Seoul 04107, Korea

[2] Research Institute for Basic Science, Sogang University, 35 Baekbeom-Ro, Mapo-Gu, Seoul 04107, Korea

[3] Research Center for Endangered Species, National Institute of Ecology, 23, Gowol-Gil, Yeongyang-eup, Yeongyang-gun, Gyeongsangbuk-do, 36531, Korea.

[¶] These authors contributed equally.

*Corresponding Author

kjung@sogang.ac.kr

Supplementary Table and Figures

Supplementary Table 1: Primers used in the mutagenesis study.

Sample	Primer forward sequence (5' to 3')	Mer
T182G	GGT CAG CGG GAT TCC CTT CG	20
F185W	GAT TCC CTG GGC CTA CA	17
T179G/T182G	GGT GGA GTC AGC GGA ATT CC	20
T179G/T182G/F185W	GGT GGA GTC AGC GGA ATT CCC TGG GCC TAC	30
P226G	GGG TGT TTA CGG GAT CGC AT	20
I227G	GGG TGT TTA CCC GGG CGC AT	20
S221G/P226G/I227G	GTT GCT CGG CTG GGG TGT TTA CGG AGG AGC ATA C	34
T179G	CTG GGG TGG AGT CAG CAC GAT TCC CT	26
S181G	CTG GGG TAC GGT CGG CAC GAT TCC CT	26
T179G/S181G	CTG GGG TGG AGT CGG CAC GAT TCC CT	26
S221G	GTT GCT CGG CTG GGG TGT TT	20

Supplementary Figure 1: Molecular docking of GR (PDB code: 6NWD[1]) and Canthaxanthin (PubChem CID:5281227[2]).

Rank	Est. Free Energy of Binding	Est. Inhibition Constant, KI	vdW + Hbond + desolv Energy	Electrostatic Energy	Total Inter-molecule. Energy	Interact. Surface
1	-5.34 kcal/mole	121.15 µM	-8.23 kcal/mol	+0.01 kcal/mol	-8.23 kcal/mol	839.543

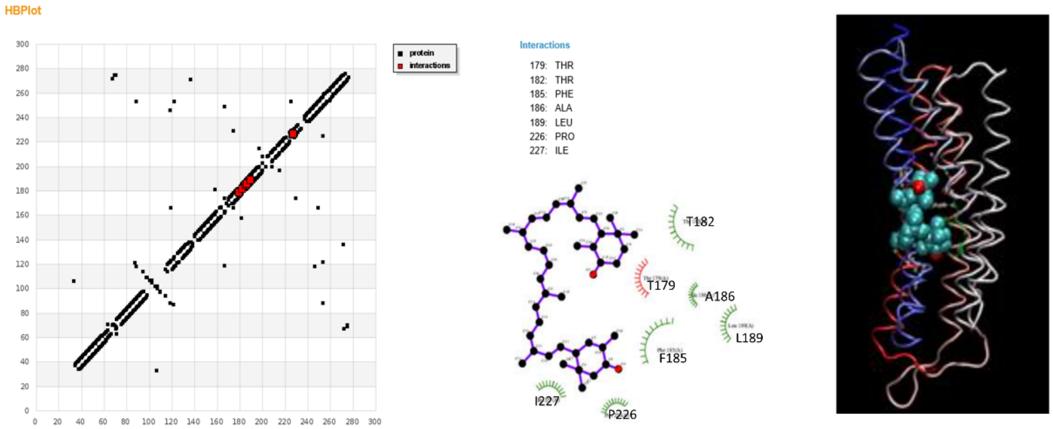

Docking calculation and the hydrogen bond interaction (HB) plot were carried out by the Molecular DockingServer[3].

Supplementary Figure 2: Absorption spectra of extracted chromophores from purified co-expression GR variants.

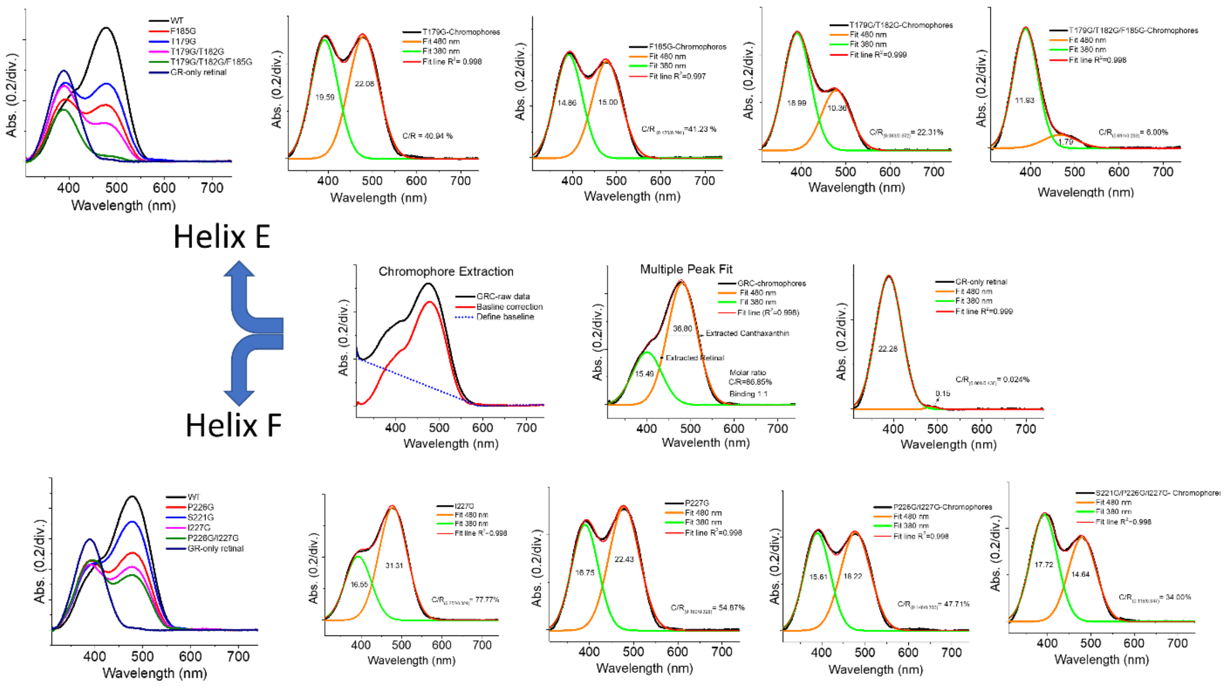

Wild-type expression with carotenoid (GRC) and only with retinal (GR) is shown in the middle panel and defines linear baseline correction, while mutation on helix E and F are shown in the upper and lower panel, respectively.

Supplementary Figure 3: Comparison of carotenoid binding kinetic in wild-type, CLM, and CAM deleted mutants for initial 22 minutes and 24 hours.

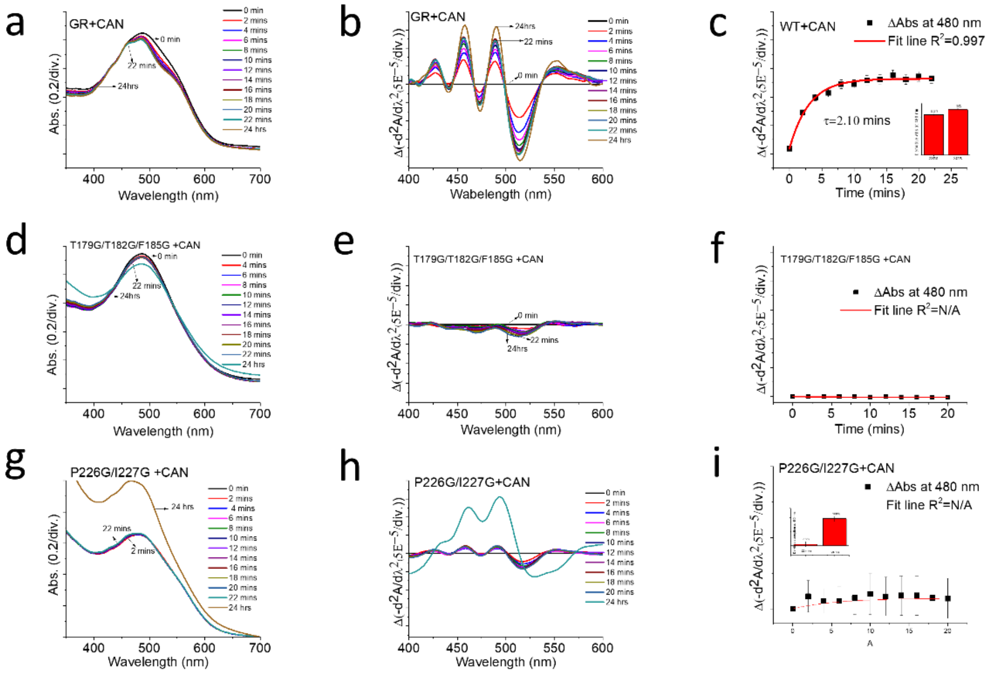

(a) Absorption spectra changes of wild-type. (b) Secondary derivative (multiply -1) of wild-type. (c) Change of derivative absorption at 480 nm, the inset bar graph is shown ~88% of carotenoid binds to GR wild-type within 22 minutes, and time constant tau=2.10 minutes. The exact order (d to f) for CLM deleted mutant (T179G/T182G/F185G) showed no binding in the first 22 minutes and after 24 hours. (g to i) CAM deleted mutant (P226G/I227G) showed about 5% binding in the first 22 minutes and undefined binding time constant, suggesting a prolonged binding rate.

Supplementary Figure 4: Secondary derivative absorption spectra of mutant, CLM, and CAM mutants (multiply -1).

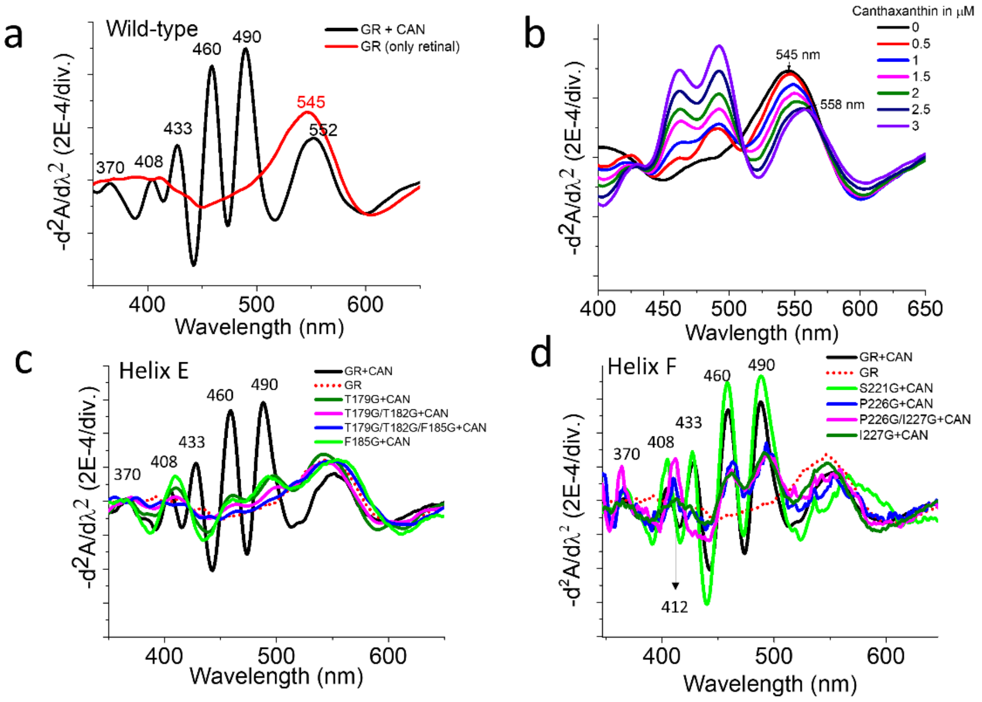

(a) The secondary derivative spectrum of wild type GR bind carotenoid showed a sharp vibrational band in a blue-green region with five absorption peaks 370, 408, 433, 460, 490 for carotenoid and 552 nm for retinal (black line), while GR with only retinal showed one peak in the green region at 545 nm. (b) Derivative absorption of reconstituted purified GR with canthaxanthin. (c) Derivative absorption of helix E mutants. (d) The secondary derivative absorption of helix F mutants.

Supplementary Figure 5: Comparison of proton pumping with and without carotenoid of GR variants.

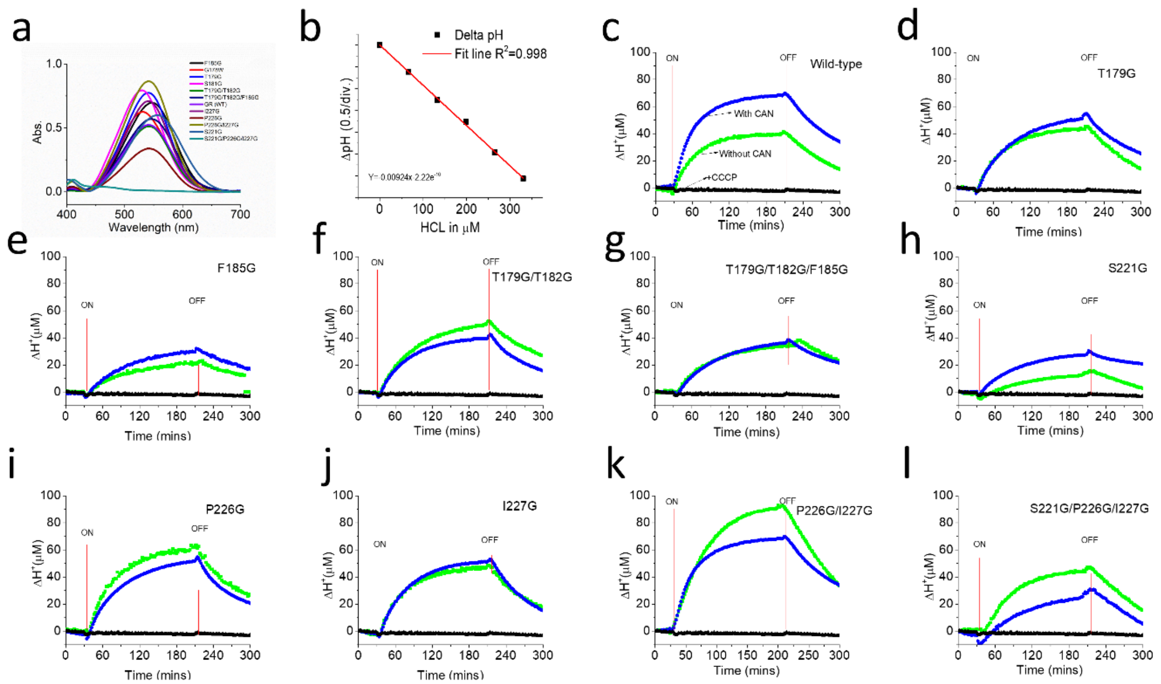

(a) UV-vis spectra of each mutant. (b) Titration of HCL to the unbuffered solution. (c-l) Comparison of proton pumping with and without carotenoid binding in wild-type and mutants.

Supplementary Figure 6: Comparison of excitation spectra for fluorescence emission of the retinal chromophore at 720 nm of GR variants.

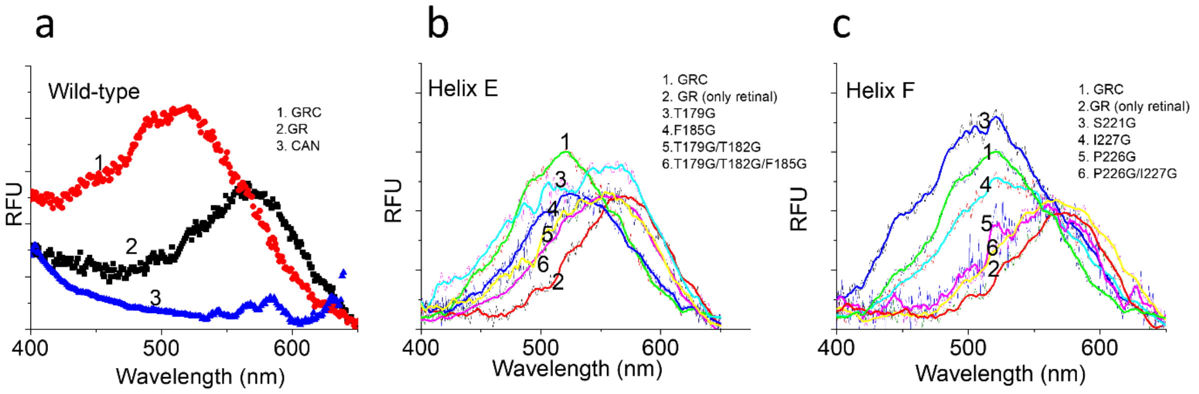

(a) GR wild-type bound carotenoid (1), with only retinal chromophore (2), and canthaxanthin (3); fluorescent intensity before baseline correction. (b) excitation spectra for fluorescence emission of the retinal chromophore at 720 nm for helix E mutants. (c) excitation spectra for fluorescence emission of the retinal chromophore at 720 nm for helix F mutants. Samples was prepared in pH 4.0 (0.02% DDM, 150 mM NaCl, 50 mM Tris) at O.D ~ 0.1[4].

Supplementary Figure 7: A single mutation replaces and mimics carotenoid binding in xanthorhodopsin.

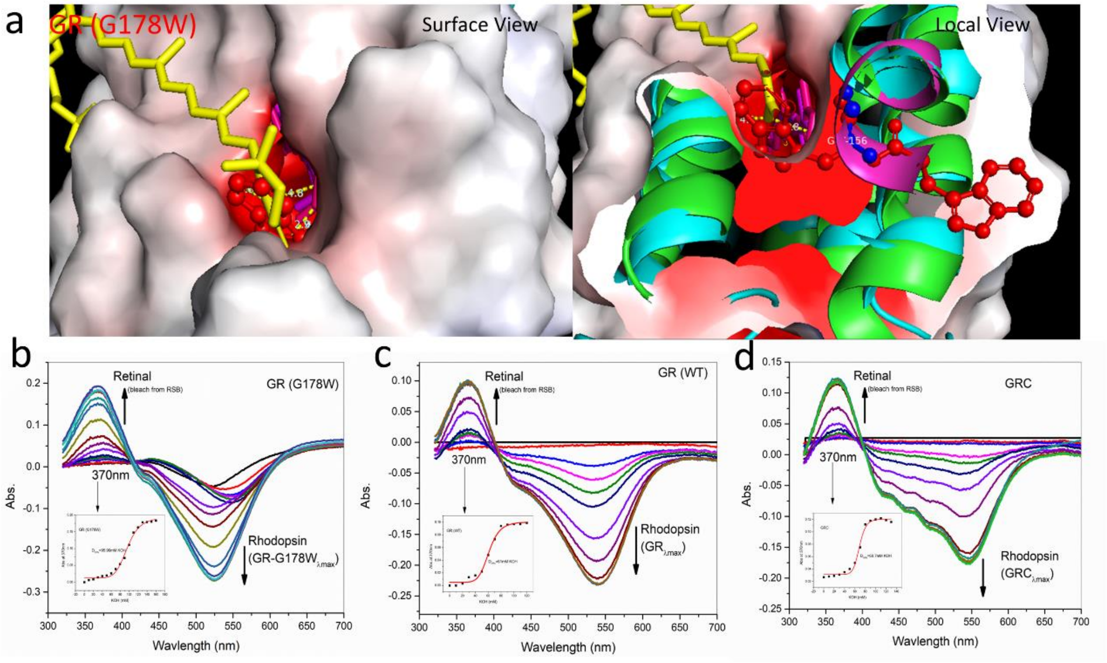

(a) The hydrophobic score was marked red with the surface and local view of GR and XR alignment. The carotenoid is marked yellow, and mutated tryptophan is marked red. As the binding of carotenoids gave the cover to a hydrophobic valley on helix E, G178W showed aromatics to be overlapped and mimic the cover upon binding to carotenoid, which then sufficiently abolished the binding of carotenoid in this mutant. (b-d) Comparison of protein stability in the exposed to high alkaline and urea, GR is one of the stable rhodopsins. So, we treat the solution with 3 M urea and titrate with KOH to test the stability of the G178W mutant, GR wild-type, and GR bound carotenoid. The results G178W showed the highest tolerance compared to the wild-type and GR bound carotenoid.

Supplementary Figure 8: The excitation of GR variants. The mutation of the carotenoid binding site showed minimal change in protein maximum absorption but significant changes in proton pumping.

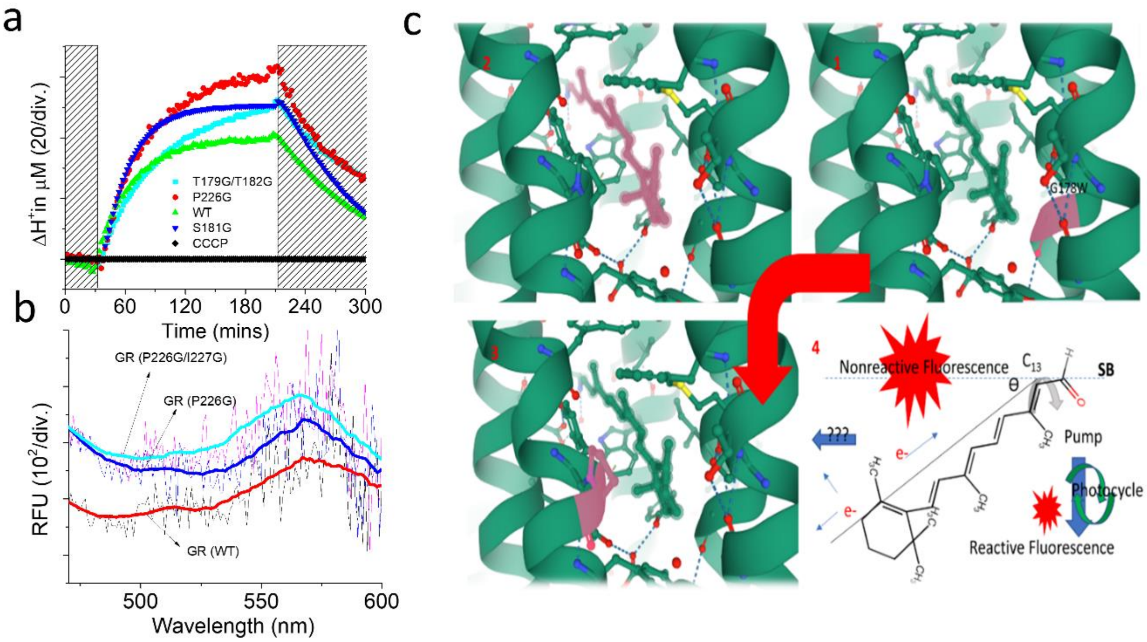

(a) excitation spectra for fluorescence emission of the retinal chromophore at 720 nm (b). A proposed scheme of excited retinal and the effect of the mutation at the carotenoid binding site (c), (1) Gly 178 position where the exciting energy of the carotenoid can be transferred to retinal, (2) excited retinal used the energy to run photo cycles and proton pumping, (3) at the same time, Pro 226 withholds retinal β-ionone ring and fastens the relaxation of excited energy, (4) the graphic of suggested structural change upon mutation while G178W triggers the change of polar environment at the position of T179, S181, and P226 might promote the excited energy transfer to SB for proton pumping, and absence of P226, then additional I227 ditag retinal β-ionone ring makes the extension of excited state life (ESL) time result in more energy transfer to SB, however, is unstable. Then the elongated ESL relaxation produced higher fluorescent in P226G and P226G/I227G.

Supplementary Figure 9: Serine 181 position is adaptive relationally to the loss of carotenoid binding. Structural change of G178W increased the distance of the polar side chain of serine 181 toward retinal, and natural homologs of the GR sequence have conserved residues at position 181

either as glycine or alanine, while the relatively the sequence with Trp178 also acquired most of the alanine in 181 positions.

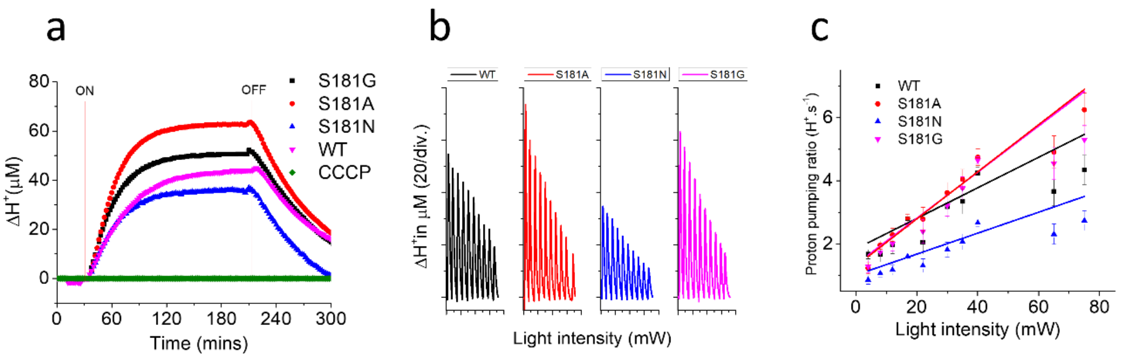

(a) Comparison of proton pumping of S181N, wild-type, S181G, and S181A confirmed that replacing the serine with smaller residues result in a better proton pump species. (b) Light intensities dependent proton pumping of the wild-type and Ser 181 mutants. (c) The fluency curve calculated from integrated proton changes in different light intensities showed that either S181G or S181A promotes proton pumping efficiency.

Supplementary Figure 10: Carotenoid binding in microbial rhodopsin conserved in other ion-pumping rhodopsin.

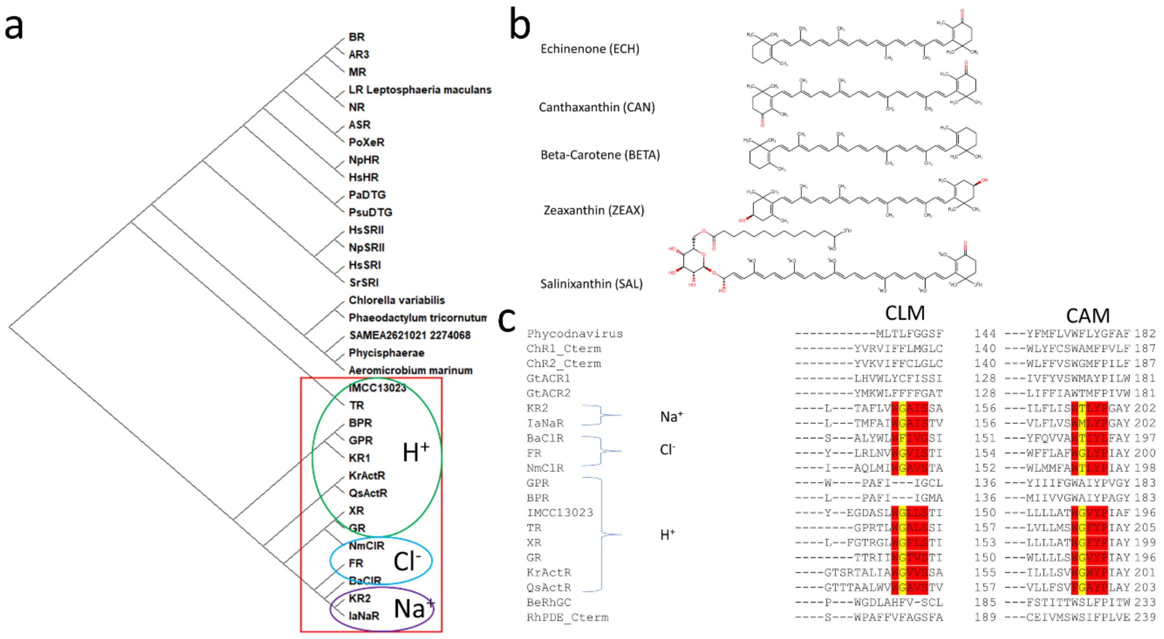

(a) The phylogenetic tree of xanthorhodopsin to other microbial rhodopsins includes proton-pumping, sodium pumping, and chloride-pumping rhodopsin. (b) Some of the known carotenoids that bind to GR (echinenone, canthaxanthin, and salinixanthin, the important 4-keto ring are conserved) versus the unbound carotenoids (beta-carotene, and zeaxanthin, which do not have the keto ring). (c) Multiple sequence alignment with carotenoid locked and aligned motifs highlighted.

Supplementary References

1. Morizumi, T. *et al.* X-ray Crystallographic Structure and Oligomerization of Gloeobacter Rhodopsin. *Sci Rep* **9**, 11283 (2019).
2. PubChem. Canthaxanthin. https://pubchem.ncbi.nlm.nih.gov/compound/5281227.
3. Bikadi, Z. & Hazai, E. Application of the PM6 semi-empirical method to modeling proteins enhances docking accuracy of AutoDock. *J Cheminform* **1**, 15 (2009).
4. Balashov, S. P. *et al.* Reconstitution of Gloeobacter Rhodopsin with Echinenone: Role of the 4-Keto Group. *Biochemistry* **49**, 9792–9799 (2010).
